# Supplementary material for: Validation of the COVID-19 Digital Health Literacy Instrument in the Italian Language: A Cross-Sectional Study of Italian University Students
Source: Int J Environ Res Public Health. 2022 May 20;19(10):6247. doi: 10.3390/ijerph19106247 (PMC9140816; doi:10.3390/ijerph19106247)
Supplement: Supplementary file 1 [file ijerph-19-06247-s001.zip › ijerph-1682555-supplementary.pdf]

Table S1. Acronyms' definition

Acronyms	Definition
HL	Health Literacy
DHL	Digital Health Literacy
DHLI	Digital Health Literacy Instrument
eHEALS	eHealth Literacy Scale
DHLIsearch	DHLI – subscale searching the web for information on COVID-19
DHLIcont	DHLI – subscale adding self-generated content on COVID-19
DHLIrely	DHLI – subscale evaluating the reliability of COVID-19-related information
DHLIrelev	DHLI – subscale determining personal relevance of COVID-19-related information
DHLIpriv	DHLI – subscale DHLI – subscale protecting privacy on the Internet
PCA	Principal Component Analysis
CFA	Confirmatory Factor Analyses
KMO	Kaiser-Meyer-Olkin test
RMSEA	Root Mean Square Error of Approximation
SRMR	Standardized Root Mean Square Residual
GFI	Goodness of Fit Index
CFI	Comparative Fit Index
NNFI	Non-normed Fit Index
PNFI	Parsimony Normed Fit Index
PGFI	Parsimony Goodness-of-Fit Index
COVID-19	Coronavirus Disease 19
WHO	World Health Organization

Table S2. COVID-19 Digital Health Literacy Instrument: English and Italian version.

English Version		Italian Version	
DHLI – information searching (DHLIsearch)<br><br>When you search the Internet for information on coronavirus or related topics, how easy or difficult is it for you to…<br>Possible responses: (1) very difficult; (2) difficult; (3) easy; (4) very easy		*DHLI – ricerca di informazioni (DHLIsearch)*<br><br>Quando cerchi su Internet informazioni sul coronavirus o argomenti relativi, quanto facile o difficile è per te…<br>Risposte possibili: (1) molto difficile; (2) difficile; (3) facile; (4) molto facile	
(DHLIsearch1)	…make a choice from all the information you find?	(DHLIsearch1)	…scegliere tra tutte le informazioni che trovi?
(DHLIsearch2)	.... use the proper words or search query to find the information you are looking for?	(DHLIsearch2)	…usare le parole o le domande di ricerca adatte per trovare le informazioni che cerchi?
(DHLIsearch3)	… find the exact information you are looking for?	(DHLIsearch3)	…trovare esattamente le informazioni che cerchi?
DHLI – adding self-generated content (DHLIcont)<br><br>When typing a message (eg. on a forum, or on a social media such as Facebook or Twitter) about the coronavirus or related topics, how easy or difficult is it for you to…<br>Possible responses: (1) very difficult; (2) difficult; (3) easy; (4) very easy		*DHLI – aggiungere contenuti propri (DHLIcont)*<br><br>Quando scrivi un messaggio (ad es. In un forum o su un social media come Facebook o Twitter) sul coronavirus o argomenti relativi, quanto facile o difficile è per te…<br>Risposte possibili: (1) molto difficile; (2) difficile; (3) facile; (4) molto facile	
(DHLIcont1)	…clearly formulate your question or health-related worry?	(DHLIcont1)	…formulare chiaramente la tua domanda o le tue preoccupazioni relative alla salute?
(DHLIcont2)	…express your opinion, thoughts, or feelings in writing?	(DHLIcont2)	…esprimere le tue opinioni, pensieri o sensazioni per iscritto?
(DHLIcont3)	…write your message as such, for people to understand exactly what you mean?	(DHLIcont3)	…scrivere il tuo messaggio in modo che le persone capiscano esattamente cosa intendi?
DHLI - evaluating reliability (DHLIrely)<br><br>When you search the Internet for information on the coronavirus or related topics, how easy is or difficult is it for you to…<br>Possible responses: (1) very difficult; (2) difficult; (3) easy; (4) very easy		*DHLI – valutare l'affidabilità (DHLIrely)*<br><br>Quando cerchi su Internet informazioni sul coronavirus o argomenti relativi, quanto facile o difficile è per te…<br>Risposte possibili: (1) molto difficile; (2) difficile; (3) facile; (4) molto facile	
(DHLIrely1)	…decide whether the information is reliable or not?	(DHLIrely1)	…decider se le informazioni sono affidabili o meno?

(DHLIrely2) …decide whether the information is written with commercial interests?	(DHLIrely2) …decider se le informazioni sono scritte con interessi commerciali (es, da persone che cercano di vendere un prodotto)?
(DHLIrely3) …check different websites to see whether they provide the same information?	(DHLIrely3) …controllare diversi siti web per vedere se forniscono le stesse informazioni?
DHLI – determining relevance (DHLIrelev)<br><br>When you search the Internet for information on the coronavirus or related topics, how easy is or difficult is it for you to…<br>Possible responses: (1) very difficult; (2) difficult; (3) easy; (4) very easy	*DHLI – valutare la rilevanza (DHLIrelev)*<br><br>Quando cerchi su Internet informazioni sul coronavirus o argomenti relativi, quanto facile o difficile è per te…<br>Risposte possibili: (1) molto difficile; (2) difficile; (3) facile; (4) molto facile
(DHLIrelev1) …decide if the information you found is applicable to you?	(DHLIrelev1) …decider se le informazioni che trovi fanno al caso tuo?
(DHLIrelev2) …apply the information you found in your daily life?	(DHLIrelev2) …applicare le informazioni che trovi nella vita di tutti i giorni?
(DHLIrelev3) …use the information you found to make decisions about your health?	(DHLIrelev3) …usare le informazioni che trovi per prendere decisioni sulla tua salute (es. sulle misure protettive, misure igieniche, vie di trasmissione, rischi e prevenzione dei rischi?
DHLI – protecting privacy (DHLIpriv)<br><br>When you post a message about the coronavirus or related topic on a public forum or social media, how often …<br>Possible responses: (1) often; (2) several times; (3) once; (4) never	DHLI – protezione della privacy (DHLIpriv)<br><br>Quando pubblichi un messaggio sul coronavirus o argomenti relativi su un forum pubblico o sui social media, quanto spesso….<br>Risposte possibili: (1) spesso; (2) più volte; (3) una volta; (4) mai
(DHLIpriv1) …do you find it difficult to judge who can read along?	(DHLIpriv1) …trovi difficile capire chi potrebbe leggerlo?
(DHLIpriv2) …do you share your own private information?	(DHLIpriv2) …condividi (volontariamente o involontariamente) le tue informazioni personali?
(DHLIpriv3) …do you share someone else's private information?	(DHLIpriv3) …condividi (volontariamente o involontariamente) le informazioni personali di qualcun altro?
